# Comparative Expression Analysis of Cytochrome P450 1A1, Cytochrome P450 1B1 and Nuclear Receptors in the Female Genital and Colorectal Tissues of Human and Pigtailed Macaque

**DOI:** 10.24947/2380-5552/2/1/120

**Published:** 2016-05-03

**Authors:** Minlu Hu, Tian Zhou, Andrew P Pearlman, Dorothy L Paton, Lisa C Rohan

**Affiliations:** 1 Department of Pharmaceutical Sciences, School of Pharmacy, University of Pittsburgh, Pittsburgh, PA; 2 Magee-Womens Research Institute, Pittsburgh, PA; 3 Department of Obstetrics and Gynecology, University of Washington, Seattle, WA

**Keywords:** CYP1A1, CYP1B1, Nuclear Receptor, Pigtailed Macaque, Female Genital Tract, Colorectal Tissue, Cancer

## Abstract

This manuscript summarizes our recent progress in examine the CYP1A1 and CYP1B1 as well as a number of nuclear receptors in the female genital and colorectal tissues of human and pigtailed macaque. Understanding the nuclear receptor mediated regulation of CYP1A1 and 1B1 expression in these tissues is necessary for identifying cancer risk factors and developing CYP1A1/1B1-targeted anti-cancer therapeutics. However, there is a lack of systematic and comparative analysis of the expression profile of CYP1A1, 1B1 and NRs in the female genital and colorectal tissues of human and clinically relevant animal models. The current study aims to fill this gap. We found CYP1A1, CYP1B1 and a number of nuclear receptors were expressed in the female genital and colorectal tissues of human and macaque. However, the mRNA level and protein localization of these CYP enzymes and NRs depended on the type of tissue examined.

Cytochrome P450 (CYP) 1A1 and CYP1B1 activate hormonal and environmental procarcinogens, and are associated with carcinogenesis in female genital and colorectal tissues. Understanding the nuclear receptor (NR) mediated regulation of CYP expression in these tissues is necessary for identifying cancer risk factors and developing CYP1A1/1B1-targeted anti-cancer therapeutics. The study aims to analyze the expression profile of CYP1A1, 1B1 and NRs in the female genital and colorectal tissues of human and pigtailed macaques. We found that compared to the liver, human CYP1A1 mRNA level in the genital and colorectal tissues was significantly lower, while the CYP1B1 level was significantly higher. CYP1A1 protein was mainly localized in the plasma membrane of the uterine and endocervical epithelial cells. The CYP1B1 protein was concentrated in the nucleus of genital and colorectal tissues. Fourteen NRs in the genital tract and 12 NRs in colorectal tissue were expressed at levels similar to or higher than the liver. The expression and localization of CYP1A1, CYP1B1, and NRs in macaque tissues were usually comparable to those of human tissues. In addition, menopause did not significantly alter the ectocervical mRNA levels of CYP1A1, CYP1B1, or NRs.

## Introducton

Cytochrome P450s (CYPs) 1A1 and 1B1 are major members of the CYP1 family, and metabolize a variety of hormonal and environmental procarcinogens [1-4]. Therefore, CYP1A1 and 1B1 play an important role in the carcinogenesis of female genital and colorectal tissues, where those procarcinogens accumulate [5-8]. The genetic polymorphisms of CYP1A1/1B1, which lead to altered metabolic activity, are associated with the risk of cancer or other premalignant diseases in the human uterus, cervix, vagina and colorectal tissue [5-9]. Due to the observed association between CYP1A1/1B1 polymorphisms and cancer risks in female genital and colorectal tissues, there has been increased interest in studying the regulation of these two enzymes in female genital and colorectal tissues. Such studies will reveal the signalling pathways of CYP1A1/1B1 regulation, and will likely contribute to the development of novel anti-cancer strategies that target the critical pathway components. In addition, such studies will help identify pathophysiological factors that lead to altered CYP1A1/1B1 expression and increased cancer risk, thus providing useful information for the prevention of female genital and colorectal cancers. Therefore, the improved understanding of CYP1A1/1B1 regulation in female genital and colorectal tissues will help cancer prevention and/or treatment in these tissues.

The nuclear receptors (NRs) are key mediators of the transcriptional regulation of CYP1A1 and 1B1. Among the reported NRs, the aryl hydrocarbon receptor (AhR) was studied in depth [10-12]. In addition, CYP1A1/1B1 can be regulated by various other NRs through direct binding to the enzyme gene regulatory elements, or through the interaction with AhR. Such NRs include pregnane X receptor (PXR), constitutive androstane receptor (CAR), vitamin D receptor (VDR), peroxisome proliferator-activated receptors (PPARs), estrogen receptors (ERs), glucocorticoid receptor (GR), retinoic acid receptors (RARs), retinoid X receptor (RXR), and nuclear factor (erythroid-derived 2)-like 2 (Nrf2) [4,13-18].

Despite extensive studies in other tissues and cell culture models, the NR-mediated regulation of CYP1A1/1B1 in female genital and colorectal tissues is poorly understood. This is due, at least in part, to the lack of a systematic evaluation of the expression profile of CYP1A1, 1B1, and NRs in the female genital and colorectal tissues of human and biologically relevant *in vivo* models. The tissue distribution, substrate specificity and functionality of CYP1A1 and 1B1 enzymes and NRs are species-specific [1,19]. In addition, although the cell culture models are powerful tools for studying NRs, significant differences in NR expression and functionality have been observed between cell cultures and *in vivo* models. Therefore, a biologically relevant model that closely mimics human biology is preferred for the study of NR-mediated CYP regulation, as long as resources permit. The pigtailed macaque has been considered as such a biologically relevant model. The morphology and physiology of macaque female reproductive and colorectal tracts are very similar to those of corresponding human tissues [20-22]. Hence, the pigtailed macaque has been used extensively in the studies of reproductive and colorectal pathology, as well as for the testing of vaginally and rectally administered drug products [20-22]. As such, it is tempting to utilize the macaque model to study NR-mediated CYP1A1 and CYP1B1 regulation in female genital and colorectal tissues. Comparative characterization of the expression profiles of CYP1A1, CYP1B1 and NRs in female genital and colorectal tissues of the human and macaque would be the first step to initiate such investigations.

In this study, we examined the mRNA levels of CYP1A1, CYP1B1 and 17 NRs relevant to CYP enzyme regulation in the endocervix, ectocervix, vagina and colorectal tissue of premenopausal women and pigtailed macaques. We also examined the protein localization of CYP1A1 and 1B1 in these tissues and compared the expression of CYP1A1, 1B1 and NRs between pre- and postmenopausal human ectocervix. To our knowledge, this is the first systematic evaluation of the CYP1A1, 1B1 and NR expression in human and macaque genital tract and colorectal tissues. This comparative analysis will inform future investigations of CYP1A1 and 1B1 regulation, and will facilitate the study of other functional genes subject to NR regulation in female genital and colorectal tracts.

## Materials and Methods

### Acquisition of Human and Pigtailed Macaque Tissues

Human genital and colorectal tissues (uterus, endocervix, ectocervix, vagina, colorectum) were obtained from women undergoing hysterectomy for benign conditions. Human liver tissues (collected as controls) were obtained from donors without hepatic malignancies. The acquisition of all human tissues was through the University of Pittsburgh Medical Center under the protocols approved by the Institutional Review Board. The three pigtailed macaques used in this study were 12.6, 18.7 and 17.6 years old, and were considered as reproductively active. The macaques were maintained in Washington National Primate Research Center at the University of Washington, in accordance with the Animal Welfare Act and the Guide for the Care and Use of Laboratory Animals. The macaque tissues (uterus, endocervix, ectocervix, vagina, colorectum, liver) were acquired through the Tissue Distribution Program, which was approved by the Institutional Animal Care and Use Committee.

### Real-Time RT-PCR

The total RNA was extracted using the Trizol reagent (Invitrogen) according to the manufacturer's instructions. The genomic DNA remaining in the total RNA preparations was removed using the Turbo DNase kit (Ambion). The reverse transcription was performed using the SuperScript III First Strand Synthesis Kit (Invitrogen). The real-time RT-PCR was conducted using the Ssofast Evergreen Mastermix (Bio-Rad), in the CFX Touch 96 thermocycler (Bio-Rad). Details concerning PCR primers can be found in Table 1. Due to the high homology between human and macaque in the mRNA sequences of CYP1A1, CYP1B1 and GAPDH, the same primers were used to detect both human and macaque genes. For the PCR programs, the initial denaturation was set at 95°C for 30 s, and 40 cycles of amplification were run at 95°C for 5 s and 60°C for 5 s. A melt curve analysis was conducted upon the completion of the amplification cycles, to ensure the specificity of the PCR reaction. The PCR efficiency was confirmed to be within the range of 90%-110%, using serially diluted cDNA standards prepared from the liver or colon tissues. The mRNA levels of CYP1A1 and CYP1B1 in a given tissue sample were normalized to the level of glyceraldehyde 3-phosphate dehydrogenase (GAPDH) using the 2^-ΔCt^ method in the same sample, and multiplied by 10^6^.

### Immunohistochemical Staining

The immunohistochemical staining was performed by the Research Histology Service of the University of Pittsburgh. The human and macaque tissues were fixed in 10% neutral-buffered formalin for no less than 24 hours, and subsequently embedded in paraffin. Sections 5 μm in thickness were made and de-paraffinized using xylene. The target antigens were retrieved after incubation with the pH 9 retrieval buffer (Dako) for 30 minutes. The slides were treated with 3% H_2_O_2_ for 5 minutes, and blocked using the Avidin block solution (Vector), Biotin block solutions (Vector), and serum, respectively. For CYP1A1 staining, the primary rabbit anti-human CYP1A1 polyclonal antibody (H-70 clone, sc-20772, Santa Cruz Biotechnology) was applied to the slides at 1:100 dilution, and incubated at 4 °C overnight. For CYP1B1 staining, the primary rabbit anti-human CYP1B1 polyclonal antibody (ab33585, Abcam) was applied at 1:750 dilution and incubated at 4 °C overnight. After the application of primary antibody, the slides were washed and incubated with the secondary antibody biotinylated goat anti-rabbit IgG (Vector) diluted in goat serum (1:200). The slides were incubated with the secondary antibody at room temperature for 30 minutes. The AEC chromogen (Skytec) was used to develop the red color on stained slides. In the negative control staining, the primary antibodies were replaced by the IgG purified from the serum of non-immunized rabbit. Human urinary bladder was used as positive control of CYP1A1. Since positive staining for CYP1B1in human ectocervix was reported previously [23], ectocervical tissue was used as the positive control for CYP1B1 in this study.

### Western blot

The human ectocervical tissue was homogenized using the Tissue Tearor homogenizer (Cole Parmer). The tissue homogenate was centrifuged at 10,000 g for 10 minutes, and the supernatant was transferred to a clean tube. The cytoplasm fraction and nucleus fraction were purified from the supernatant using the Nuclear Extraction Kit (Millipore). The total protein concentrations in the cytoplasm and nucleus preparations were measured using the Micro BCA Protein Assay Kit (Thermo). The purified fractions were then subjected to the sodium dodecyl sulfate polyacrylamide gel electrophoresis (SDS-PAGE), using the Mini-PROTEAN precast gels (Bio-Rad). After electrophoresis, the gels were electro-blotted onto a nitrocellulose membrane (Invitrogen) in an iBlot Dry Blotting System (Invitrogen). All the subsequent procedures were performed at room temperature. The blotted membranes were blocked in milk for 1 hour, then incubated with the primary antibodies of CYP1B1 (Abcam, ab33585) and GAPDH (Santa Cruz, sc-48167 for 1 hour. The dilutions were 1: 1000 for CYP1B1 antibody and 1: 500 for GAPDH antibody. The HRP-conjugated goat anti-rabbit IgG (1:2000, Cell Signaling #7074s) were incubated with the membrane for 1 hour. The Pierce ECL plus Western blotting substrate (Thermo) was applied afterwards to detect HRP activity, and the chemiluminescence images were taken using the ChemiDoc MP imaging system (Bio-Rad).

### Statistical Methods

The comparison in the mRNA levels between different types of tissue was performed using the one way analysis of variance (ANOVA) with Bonferroni post-hoc test. *P* < 0.05 was considered as statistically significant.

## Results

### The Mrna Levels of CYP1A1, 1B1 in the Genital Tract and Colorectal Tissues of Premenopausal Women and Macaques

**Figure 1:**
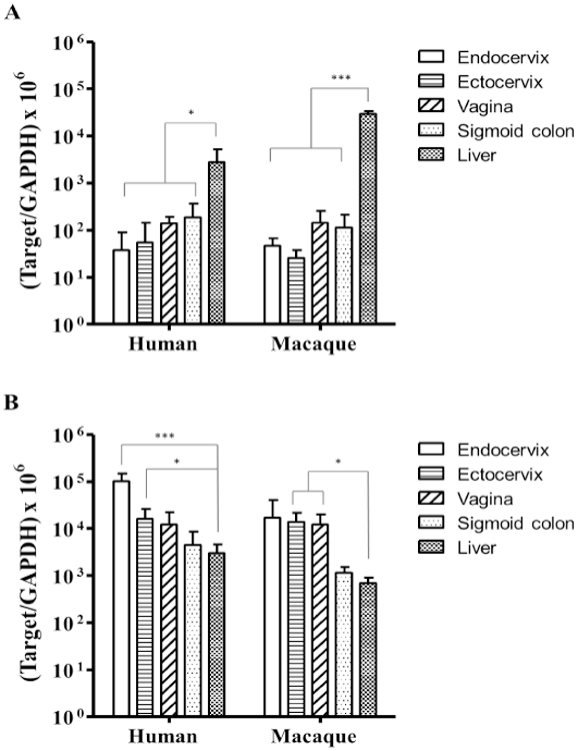
The mRNA levels of CYP1A1 (**A**) and CYP1B1 (**B**) in the genital tract and colorectal tissue of premenopausal women and pigtailed macaques. The tissues for endocervix, ectocervix, vagina, sigmoid colon and liver were from human donors (endocervix: n= 4; ectocervix: n= 6; vagina: n= 5; sigmoid colon: n= 5; liver: n= 6). For each type of tissue, 50-200 mg of tissue was collected from each donor and used for RNA extraction and subsequent RT-PCR analysis. The threshold cycle numbers (Ct) of enzymes and GAPDH of each sample were measured in triplicates, and the average Ct was used to reflect the Ct of a tested gene. All tested gene levels were generated using 2^-ΔCt^ method and normalized to GAPDH and multiplied by 10^6^. The data shown represent mean ± standard deviation of all samples. (□): endocervix, ( 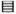): ectocervix, ( 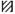): vagina, ( 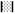): colorectal tissue, ( 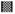): liver. *, p<0.05; ***, p<0.001

In human tissues, the CYP1A1 mRNA levels in endocervix, ectocervix, vagina and sigmoid colon were significantly lower than their levels in the liver in which the CYP enzyme expression and functionality have been well studied, while there was no significant difference among endocervix, ectocervix, vagina and colon (Figure 1A). The CYP 1B1 mRNA level in endocervix was significantly higher than that in any other type of tissue, while there was no difference among ectocervix, vagina, sigmoid colon and liver (Figure 1B). Although CYP1A1 and 1B1 were considered to metabolize similar pools of substrates, their relative mRNA levels were different from each other, and were tissue-dependent. The mRNA levels of CYP1A1 and 1B1 were similar in human liver, while the CYP1B1 was expressed at significantly higher level compared to 1A1 in the female genital tract and colonic tissues (Figure 1, A and B).

In macaque tissues, similar mRNA expression patterns were observed for both CYP1A1 and 1B1 (Figure 1, A and B). However, differences were also observed between macaque and human. The CYP1A1 level in macaque liver was significantly higher than the liver CYP1B1 level (Figure 1, A and B). In addition, the CYP1B1 level was not significantly different among all the types of macaque tissue examined (Figure 1B).

### The Protein Localization of CYP1A1 and 1B1 in the Genital Tract and Colorectal Tissues of Premenopausal Women and Macaques

**Figure 2:**
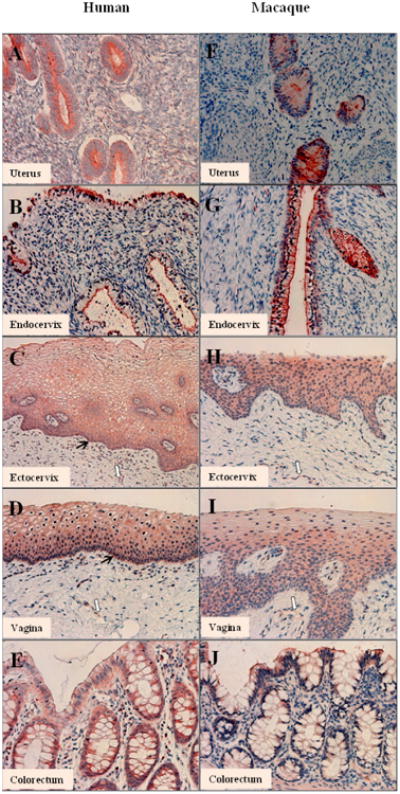
Localization of CYP1A1 protein in the genital tract and colorectal tissue of premenopausal women and pigtailed macaques. **A-E**: human uterus, endocervix, ectocervix, vagina and colorectal tissue. **F-J**: macaque uterus, endocervix, ectocervix, vagina and colorectal tissue. Black arrow: basal layers of the squamous epithelium, white arrows: vascular endothelial cells. Scale bar: 50μm for all.

In human uterus and endocervix, the CYP1A1 protein was localized on the plasma membrane of the glandular (columnar) epithelial cells, as revealed by the immunohistochemical staining (Figure 2, A and B). In human ectocervix and vagina, weak cytoplasmic staining of CYP1A1 can be found in the basal layers of the squamous epithelium (Figure 2, C and D, black arrow).

However, the positive staining pattern obtained for CYP1A1 was not confirmed when western blot was performed on the tissue fraction extracted from the ectocervix, which might due to the low protein concentration. In human sigmoid colon, the staining was overall weak on the luminal epithelial cells. The signal appeared stronger on the plasma membrane of the glandular epithelial cells (goblet cells) (Figure 2E). The staining patterns of CYP1A1 in macaque genital tract tissues were very similar to those of human tissues (Figure 2, F-I). Both human and macaque ectocervix and vagina appeared to have some positive staining on the vascular endothelial cells (Figure 2, C, D, H and I, white arrow). In addition, the staining of the glandular epithelial cells in macaque colorectum was very weak (Figure 2J). It should be noted that, due to limited patient resources, sufficient quantity of human uterus samples were not available to perform transcript quantification. Theuterus was solely investigated using immunohistochemistry staining to study the CYP1A1 and CYP1B1 localization profile in the tissue.

**Figure 3:**
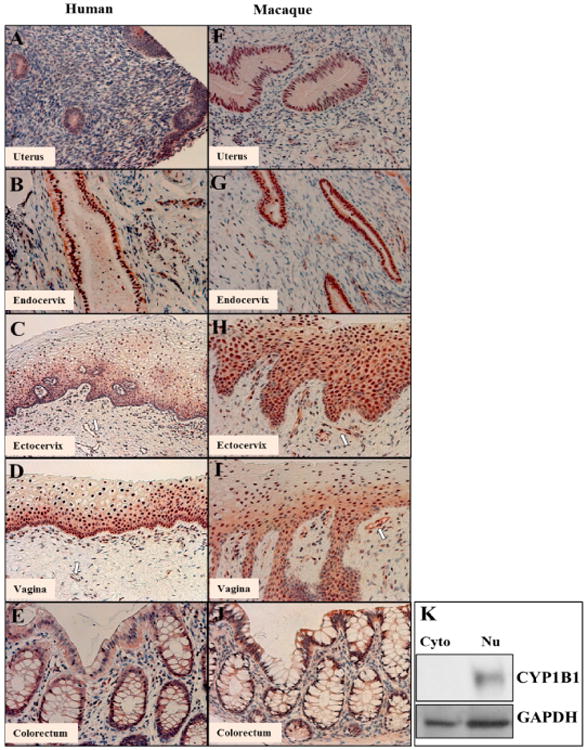
Localization of CYP1B1 protein in the genital tract and colorectal tissue of premenopausal women and pigtailed macaques. **A-E**: human uterus, endocervix, ectocervix, vagina and colorectal tissue. **F-J**: macaque uterus, endocervix, ectocervix, vagina and colorectal tissue. White arrows: vascular endothelial cells. Scale bar is 50 μm for A, B, D, E, F, G, I, H, and J; 100 μm for C. **K**: Western blot analysis of CYP1B1 in cytoplasmic and nuclear fractions from human premenopausal ectocervix. Upper panel: CYP1B1; lower panel: GAPDH as internal reference; Cyto: cytoplasmic fraction; Nu: nuclear fraction.

In human genital tract tissues, weak CYP1B1 staining was observed in the glandular (columnar) epithelial cells in the uterus (Figure 3A). Intense staining of the nucleus was observed in the glandular epithelial cells of endocervix (Figure 3B), as well as throughout the entire depth of the squamous epithelium of the ectocervix and vagina, not just the basal layers (Figure 3, C and D). In human sigmoid colon, the CYP1B1 staining was primarily found in the epithelial cells (Figure 3E). Interestingly, the CYP1B1 staining was concentrated in the nucleus and distinguished from the surrounding cytoplasm. This nucleus distribution pattern could be clearly observed in human endocervix, ectocervix and vagina (Figure 3, B-D). The nucleus localization of CYP1B1 in the lower genital tract tissues was confirmed in Western blot, using human ectocervix as the representative genital tract tissue. As shown in Figure 3K, the nucleus fraction extracted from the ectocervical tissue showed a much more intense band, compared to the cytoplasmic fraction prepared from the same type of tissue. In macaque genital tract tissues, the staining patterns of CYP1B1 were very similar to those of human tissues (Figure 3, F-I). However, the macaque uterus appeared to have stronger staining than human uterus (Figure 3F). In addition, similar staining of CYP1B1 was observed in the macaque colorectum compared to human tissue (Figure 3J).

### The Mrna Levels of Nuclear Receptors in the Genital Tract and Colorectal Tissues of Premenopausal Women and Macaques

**Figure 4:**
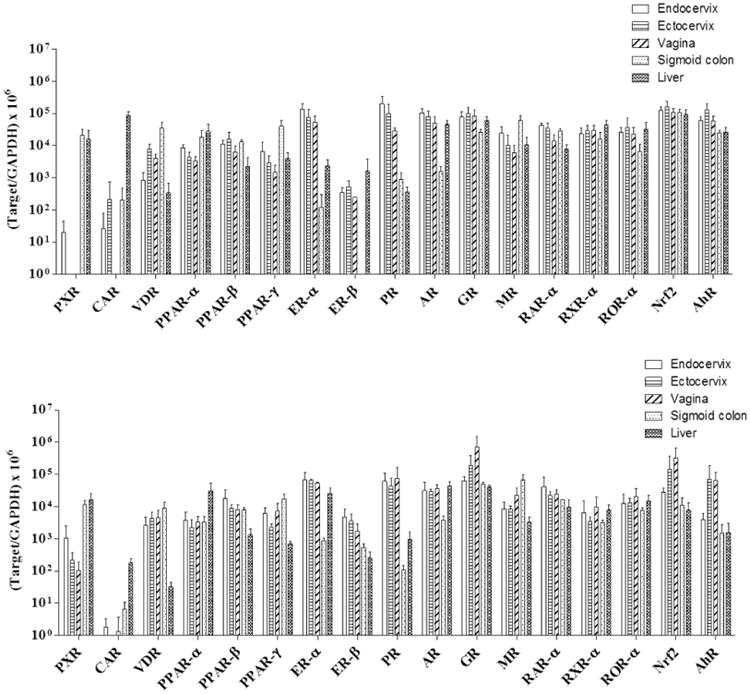
The mRNA levels of 17 nuclear receptors in the genital tract and colorectal tissue of premenopausal women (**A**) and pigtailed macaques (**B**). For human tissues, endocervix: n= 4; ectocervix: n= 6; vagina: n= 5; sigmoid colon: n= 5; liver: n= 6. For macaque tissues, n=3 for all tissue types. For each type of tissue, 50-200 mg of tissue was collected from each donor and used for RNA extraction and subsequent RT-PCR analysis. The threshold cycle numbers (Ct) of enzymes and GAPDH of each sample were measured in triplicate, and the average Ct was used to reflect the Ct of a tested gene. All tested gene levels were generated using 2^-ΔCt^ method and normalized to GAPDH and multiplied by 10^6^. The data shown represent mean ± standard deviation of all samples.

As shown in Figure 4, multiple NRs were highly expressed in human genital tract and colonic tissues compared to their levels in human liver. The mRNA levels of VDR, PPAR-β, PPAR-γ, ER-α, ER-β, PR, AR, GR, MR, RAR-α, RXR-α, ROR-α, Nrf2 and AhR in human genital tract tissues were similar or higher than their levels in human liver. In contrast to the highly expressed NRs, the mRNA levels of PXR, CAR, PPAR-α were not detectable or significantly lower in the genital tract tissues compared to the liver levels. In human sigmoid colon, the PXR, VDR, PPAR-β, PPAR-γ, PR, GR, MR, RAR-α, RXR-α, ROR-α, Nrf2 and AhR displayed similar or higher mRNA levels compared to their levels in liver. However, the mRNA levels of CAR, PPAR-α, ER-α, ER-β, AR in human colon were significantly lower than those in the liver. There was no significant difference in NR expression among different parts of the human lower genital tract (endocervix, ectocervix, and vagina). In the macaque genital tract and colorectum, the NR mRNA levels relative to macaque liver were generally similar to those in human tissues. However, subtle differences did exist between the two species. The macaque colorectal ER-β level was lower than that of human colon. In addition, the expression levels of GR, Nrf2 and AhR in macaque endocervix appeared to be lower than that of the macaque ectocervix and vagina, however statistically significant differences were not observed among these different parts of macaque genital tract.

### The Effect of Menopause on the Expression of CYP1A1, CYP1B1 and Nrs in Human Ectocervix

**Figure 5:**
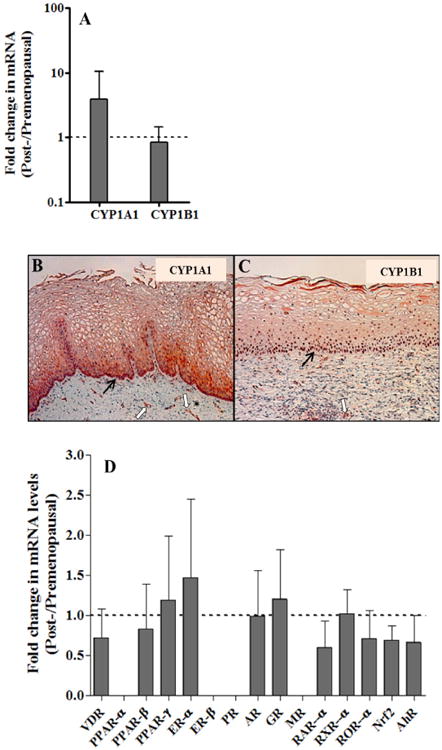
The effect of menopause on the expression of CYP1A1, CYP1B1, and NRs in human ectocervix. **A**: fold change in mRNA levels of CYP1A1 and CYP1B1 in human ectocervix collected from pre- (n=6) and post-menopausal women (n=6). **B and C**: protein localization of CYP1A1 (**B**) and CYP1B1 (**C**) in postmenopausal human ectocervix. Black arrow: basal layers of the squamous epithelium, white arrows: vascular endothelial cells. Scale bar is 100 μm for B and C. **D**: fold changes in mRNA expression of 17 NRs in human ectocervix (n=6). For A and D: The threshold cycle numbers (Ct) of enzymes and GAPDH of each sample were measured in triplicate, and the average Ct was used to reflect the Ct of a tested gene. All tested gene levels were generated using 2^-ΔCt^ method and normalized to GAPDH and multiplied by 10^6^. The data shown represent mean ± standard deviation of all samples. (□): endocervix, ( 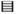): ectocervix, ( 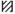): vagina, ( 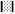): colorectal tissue, ( 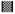): liver.

The effect of menopause was examined in the postmenopausal human ectocervix. Compared to the premenopausal tissues, the CYP1A1 mRNA level appeared to be higher in the postmenopausal tissues, but no statistical significance was observed. The mRNA levels of CYP1B1 and detectable NRs were not significantly different between pre- and postmenopausal ectocervical ectocervix (Figure 5). However, the CYP1A1 and 1B1 protein in the postmenopausal tissues appeared to be more condensed in the basal layers of the epithelial cells, compared to premenopausal tissues.

## Discussion

The expression and localization of CYP1A1, 1B1 and NRs reported in this study were in line with previously published studies by our group and other researchers [5,24-27]. To our knowledge, this is the first systematic evaluation of the expression profile of CYP1A1, 1B1 and multiple CYP-related NRs in human and macaque female genital and colorectal tissues. The information revealed in this study will provide useful information for the future research of CYP1A1, CYP1B1, and NRs in female genital and colorectal tracts.

The expression and localization profile of the CYP1A1 and 1B1 in human tissues has implications for the future research of these two enzymes in many aspects. CYP1B1 appeared to be the most important member of CYP1 family in female lower genital tract [1]. In this study, the CYP1B1 expression level was found to be significantly higher than the CYP1A1 level in the female genital tract and colorectal tissue in both human and macaque. CYP1A2 expression was not detectable in pooled human ectocervical tissues as reported in our previous publication [27]. Taken together, these observations suggested that CYP1B1 appeared to be the most important member for the CYP1 enzyme activities in female genital tract. In addition, the tissue-dependent subcellular localization patterns of CYP1A1 and 1B1 indicate that these enzymes may target different subcellular compartments in different types of tissues. The plasma membrane localization of CYP1A1 in the endocervix was not observed before, however the plasma membrane localization of other CYP isoforms, including CYP1A2, CYP2B has been reported [21,22,28]. Marie-Anne et al. studied CYP1A2 localization in rat hepatocytes and found that newly synthesized CYP1A2 followed the intracellular vesicular flow to the plasma membrane, and that the plasma membrane CYPs were mainly located on the external surface [21]. This localization indicates that the major metabolic site of CYP1A1 in the endocervix might be the plasma membrane, and those substrates that are preferentially distributed into the lipid bilayer membrane may be subject to most extensive metabolism by CYP1A1. Although the majority of the CYPs are expressed in the cytoplasm, Muskhelishvili. et al has reported that the CYP1B1 protein is located in the nucleus, in both neurons and ectocervix [29]. This finding correlates with those of this study, in that CYP1B1 was found to be concentrated in the nucleus of endocervical, ectocervical, and vaginal tissues. This indicates that, among all the substrates, those that can enter the nucleus would be preferentially metabolized by CYP1B1 in the female lower genital tract. However, Carnell et al claimed that no evidence was obtained for CYP1B1 expression in the nucleus with protein expression present exclusively in the cytoplasm of the prostate carcinoma cells [30]. This discrepancyin CYP1B1 protein subcellular localization may be due to the differences in CYP1B1 physiologic function between tissues. Another possibility is the specificity of the antibody. The CYP1B1 antibodies used in published reports came from various sources and target different epitopes. It is possible that different levels of antibody specificity may contribute to the differences observed in localization patterns. The expression profiling of multiple NRs has provided clues for the study of the regulation mechanisms of CYP1A1 and 1B1 in the female lower genital tract and colorectal tissues. The xenobiotic sensors PXR and CAR can respond to various therapeutic drugs [31], and their agonists could induce CYP1A1/1B1 regulation at mRNA level [4,13]. If these NRs are expressed in the female genital tract and colorectal tissues, then the drug-induced CYP1A1/1B1 interaction through these NRs would be possible. Steroid hormones, oxidative stress, and aryl hydrocarbon molecules could induce the CYP1A1/1B1 through binding to and activating the corresponding NRs [10-12, 14, 17, 32,33]. In this study, the xenobiotic sensors PXR, CAR, and PPAR-α were not detectable or were significantly lower in the female genital tract than in the liver, indicating that CYP1A1/1B1 was not likely to be regulated by concomitantly administered therapeutic drugs that can bind to these NRs in female reproductive tissues. The high expression of the NRs for hormone, oxidative stress and aryl aromatic hydrocarbons highlighted the possibility that their ligands may play a role in the observed up- or down-regulation of CYP1A1/1B1. Compared to the female genital tract, the colorectal tissue displayed a different expression profile of the same panel of NRs, indicating that colorectal CYP1A1/1B1 regulation mechanisms may be different from the female reproductive tract. Thus, we sought to examine the effect of menopause on CYP1A1 and 1B1 expression. Although the CYP1A1 and 1B1 mRNA levels were not significantly different in postmenopausal ectocervix, the protein localization appeared to be altered in postmenopausal tissues. Future investigations on the factors that can modulate CYP1A1/1B1 expression and activity are warranted, for better delineation of the CYP1A1/1B1 functionality in reproductive and colorectal pathology, as well as for anticancer pharmacology. It should be noted that, in addition to age and menopausal status, which were the only available patient data for the specimens procured for these studies, other factors such as stage of the sexual cycle, ethnic group, and clinical history may impact the findings presented. The impact of these additional factors should be evaluated in the future studies.

The comparison between human and macaque will guide the rational selection of preclinical models in future study of CYP1A1, 1B1 and NRs. Since the pigtailed macaque showed almost identical expression patterns of the CYP1A1, 1B1 and NRs in the female genital tract and colorectal tissues, the macaque is a good model of choice in future investigations. Besides CYP1A1 and 1B1, the highly expressed NRs reported in this study regulate various other metabolizing enzymes and transporters. Therefore, the comparison of NR expression revealed in this study is also helpful for other research endeavors that aim to study the regulation mechanism of hundreds of NR downstream genes that play important roles in disease occurrence, progression, prevention, and treatment. The difference observed in mRNA does not necessarily reflect a difference in activity. Additionally, although compared to other animal models, such as the mouse or rabbit, the macaque is considered a more clinically relevant model for the female genital tract in pharmaceutical research; it is possible that enzymes involved in the metabolism of drugs in humans might perform differently in the macaque. Further work is needed to investigate whether there are interspecies difference at both a protein and activity level.

Taken together, the discoveries obtained from this study will likely facilitate the examination of the regulation of CYP1A1 and 1B1 mediated by NRs, which will in turn lead to the development of novel approaches that utilize CYP1A1 and 1B1 in the treatment or prevention of various diseases in the female genital and colorectal tissues.

In Conclusion, CYP1A1, CYP1B1 and a number of NRs were expressed in the female genital and colorectal tissues of human and macaque. However, the mRNA level and protein localization of these CYP enzymes and NRs depended on the type of tissue examined. The high expression levels of the CYP-relevant NRs in the female genital and colorectal tissues provided clues for the study of CYP1A1 and 1B1 regulation in these tissues. The resemblance between human and pigtailed macaque in the expression patterns of CYP1A1, 1B1, and NRs suggests the utility of the macaque model for the future studies.

## Supplementary Material

**Supplementary Table 1:** Information of the human tissue donors
**Supplementary Figure 1:** Representative negative controls for CYP1A1 (A-D) and CYP1B1 (E-H). A-D: human uterus, endocervix, ectocervix, colon. E-H: human uterus, liver, ectocervix, colon. Scale bar is 50 μm for A, B, C, D, F, and H; 100 μm for E; 200 μm for G.

## Figures and Tables

**Table 1:** Information concerning primers used for the real-time RT-PCR of CYP1A1, CYP1B1, nuclear receptors (NRs), and GAPDH in human and macaque tissues.

Common Gene name	Gene Bank accession number	Primer sequences 5′ to 3′
CYP1A1	NM_000499	Forward: TCGGCCACGGAGTTTCTTC
		Reverse: GGTCAGCATGTGCCCAATCA
CYP1B1	NM_000104	Forward: AAGTTCTTGAGGCACTGCGAA
		Reverse: GGCCGGTACGTTCTCCAAAT
PXR	NM_022002	Forward: GGCCACTGGCTATCACTTCAA
		Reverse: TTCATGGCCCTCCTGAAAA
CAR	NM_001077474	Forward: GATGCTGGCATGAGGAAAGAC
		Reverse: TTGCTCCTTACTCAGTTGCAC
VDR	NM_001017536	Forward: TCTCCAATCTGGATCTGAGTGAA
		Reverse: GGATGCTGTAACTGACCAGGT
PPAR-α	NM_005036	Forward: ATGGTGGACACGGAAAGCC
		Reverse: CGATGGATTGCGAAATCTCTTGG
PPAR-β	NM_177435	Forward: TCACACAGTGGCTTCTGCTC
		Reverse: TGAACGCAGATGGACCTCTA
PPAR-γ	NM_138711	Forward: AAGGCCATTTTCTCAAACGA
		Reverse: GAGAGATCCACGGAGCTGAT
ER-α	NM_000125_	Forward: ATGATCAACTGGGCGAAGAG
		Reverse: CAGGATCTCTAGCCAGGCAC
ER-β	NM_001214902	Forward: TCCATCGCCAGTTATCACATCT
		Reverse: CTGGACCAGTAACAGGGCTG
PR	M15716	Forward: GTCAGTGGGCAGATGCTGTA
		Reverse: TGCCACATGGTAAGGCATAA
AR	NM_000044	Forward: TTGTGTCAAAAGCGAAATGG
		Reverse: AGTCAATGGGCAAAACATGG
GR	NM_001204264	Forward: ACAGCATCCCTTTCTCAACAG
		Reverse: AGATCCTTGGCACCTATTCCAAT
MR	NM_000901	Forward: GAAGTGATGGGTATCCGGTC
		Reverse: TTTGAAGGTCTTGAAGATCCAG
RAR-α	NM_000964	Forward: AAGCCCGAGTGCTCTGAGA
		Reverse: TTCGTAGTGTATTTGCCCAGC
RXR-α	NM_002957	Forward: GACGGAGCTTGTGTCCAAGAT
		Reverse: AGTCAGGGTTAAAGAGGACGAT
ROR-α	NM_134261_	Forward: ACTCCTGTCCTCGTCAGAAGA
		Reverse: CATCCCTACGGCAAGGCATTT
Nrf2	NM_006164_	Forward: ACACGGTCCACAGCTCATC
		Reverse: TCTTGCCTCCAAAGTATGTCAA
AhR	NM_001621_	Forward: TCAGTTCTTAGGCTCAGCGTC
		Reverse: AGTTATCCTGGCCTCCGTTT
GAPDH	NM_001256799	Forward: GGAGCGAGATCCCTCCAAAAT
		Reverse: GGCTGTTGTCATACTTCTCATGG
