# Supplementary material for: Comparative Expression Analysis of Cytochrome P450 1A1, Cytochrome P450 1B1 and Nuclear Receptors in the Female Genital and Colorectal Tissues of Human and Pigtailed Macaque
Source: BAOJ Pharm Sci. Author manuscript; Available in PMC 2017 Dec 20. (PMC5737814; doi:10.24947/2380-5552/2/1/120)
Supplement: Supplementary file 1 — Supplementary Table 1: Information of the human tissue donors Supplementary Figure 1: Representative negative controls for CYP1A1 (A-D) and CYP1B1 (E-H). A-D: human uterus, endocervix, ectocervix, colon. E-H: human uterus, liver, ectocervix, colon. Scale bar is 50 μm for A, B, C, D, F, and H; 100 μm for E; 200 μm for G. [file NIHMS896826-supplement-supplement_1.pdf]

Supplementary Table 1: Information of the human tissue donors

Patient #	Tissue type	Age/Age range	Gender
1	Endocervix	35-40	Female
2	Endocervix	20-25	Female
3	Endocervix	40-45	Female
4	Endocervix	25-30	Female
5	Endocervix	45-50	Female
6	Ectocervix	40-45	Female
7	Ectocervix	45-50	Female
8	Ectocervix	35-40	Female
9	Ectocervix	40-45	Female
10	Ectocervix	45-50	Female
11	Ectocervix	40-45	Female
12	Ectocervix	60-65	Postmenopausal Female
13	Ectocervix	65-70	Postmenopausal Female
14	Ectocervix	65-70	Postmenopausal Female
15	Ectocervix	70-75	Postmenopausal Female
16	Ectocervix	65-70	Postmenopausal Female
17	Ectocervix	70-75	Postmenopausal Female
18	Vagina	40-45	Female
19	Vagina	45-50	Female
20	Vagina	45-50	Female
21	Vagina	45-50	Female
22	Vagina	45-50	Female
23	Liver	30	Female
24	Liver	35	Female
25	Liver	15	Female
26	Liver	65	Male
27	Liver	49	Female
28	Liver	23	Male
23	Sigmoid colon	70-79	Female
24	Sigmoid colon	70-79	Female
25	Sigmoid colon	40-49	Female
26	Sigmoid colon	20-29	Female
27	Sigmoid colon	50-59	Female

incubated with the secondary antibody at room temperature for 30 minutes. The AEC chromogen (Skytec) was used to develop the red color on stained slides. In the negative control staining, the primary antibodies were replaced by the IgG purified from the serum of non-immunized rabbit. Human urinary bladder was used as positive control of CYP1A1. Since positive staining for CYP1B1in human ectocervix was reported previously [23], ectocervical tissue was used as the positive control for CYP1B1 in this study.

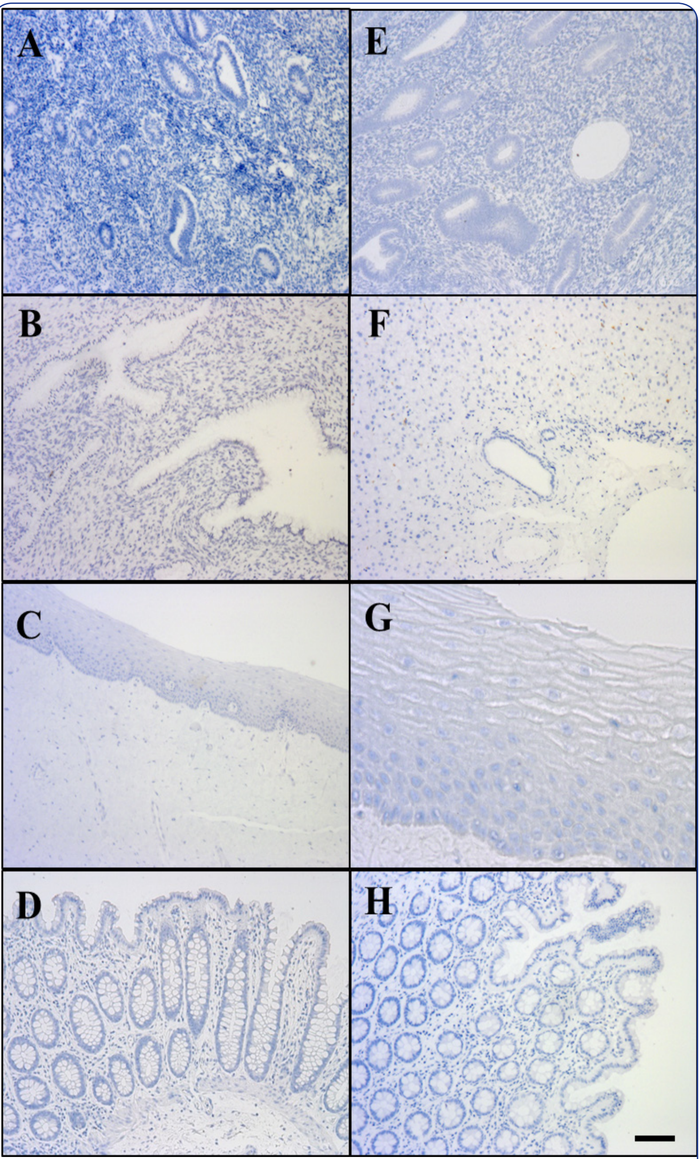

Supplementary Figure 1: Representative negative controls for CYP1A1 (A-D) and CYP1B1 (E-H). A-D: human uterus, endocervix, ectocervix, colon. E-H: human uterus, liver, ectocervix, colon. Scale bar is 50 µm for A, B, C, D, F, and H; 100 µm for E; 200 µm for G.
